# Annotations

**Published:** 1898-04-16

**Authors:** 


### Annotations.

### Middle-class Hospitals.

A lady, apparently a nurse, has sent us a circular advocating the establishment in London of a hospital for middle-class patients to be attended by their own doctors, who would have to pay a moderate residential fee. About 20 years ago this question was fully discussed and considered by the medical profession and the public. The Home Hospitals Association for Paying Patients was incorporated, and a pay hospital was opened at Fitzroy House, Fitzroy Square, which has been entirely successful. The Home Hospitals Association has power under its articles to open cheaper hospitals for middle-class patients, but a full consideration of the matter, backed by thorough knowledge of the circumstances which have to be faced, has resulted in the decision not to take any such step. If hospital accommodation is ever to be provided satisfactorily for a class of patients who pay less than a remunerative rate for their treatment when ill, it must be in paying annexes, or pay wards, in connection with the large general hospitals. There can be no doubt that any general hospital in London which had the courage to establish such a pay wing would be able to work it with great advantage to the community and adequate benefit to its finances. Fitzroy House is a pay hospital pu [illegible]nd simple, and it would be to the advantage of everybody for a bridge in the shape of pay wards to be built to connect it with the great general hospitals, so that all classes of the community might be adequately provided for in the time of sickness. Any such annexe must be separate from the establishment in which non-paying patients are treated; it must afford patients the opportunity of being attended, if they wish, by their own doctors; and it would tend to greatly improve the quality of nurse-training by enabling probationers to be thoroughly taught the duties of a private nurse. But to attempt to set up a hospital for middle-class patients as a separate institution must result in ultimate disappointment and pecuniary loss.

### A Free Electrical Hospital

From the evidence given at an inquest held last Monday, at the Kensington Town Hall, it is clear that quacks are as plentiful as ever, and that the bald impudence of the particular sort of quackery which happens to be in vogue is not the slightest bar to its acceptance by a confiding public. Here we have a man calling his house a hospital, and so deluding the public into the belief that it is a reputable establishment, when it is not a hospital at all, but a mere private venture, where, in the words of the "professor" by whom it is run, "the patients paid what they liked and the staff were paid out of it." Then this precious "hospital" professed to treat all sorts of diseases, even consumption, by electricity! Sham nurses also—women who confessed that they had received no hospital training except what they had got from the "professor"—were sent out to apply—of all things in the world—the X rays. Not that they ventured to attempt a photograph with them; that would have been too palpably to court failure. No; there is something far better than skiagraphy to be got out of the X rays. They are beautifully occult, and exactly suit a gullible public; so this electrical hospital sends out these "nurses" with something that it calls an X-ray apparatus for the treatment of disease! As the "professor" says, it is "very mild." He also says that his electricity is "very useful in cases of consumption." Surely all this is absolute false pretence, yet it goes on under the noses of the police, and poor people pay their money for this quackery, and are led to think that they are going to a hospital! More strange still, there seem to have been persons connected with this curious establishment who ought to have known better; for this man, who confessed that he not only had no medical qualification, but that he had not even had any medical training, and that, for all his positiveness about the treatment of disease, he had never seen a post-mortem, was able to boast that his "hospital" was supported by various people of position; a judge, a lady of title, a general, and "nine members of a distinguished Cabinet Minister's family" being trotted out as supporters of this pretentious quackery! People pretend often enough that education is to reform the world. But neither education nor position seems to be proof against being deluded by the most outrageous quackery and imposture. Nothing but the firm hand of the law is of any use against fortune-telling, electrical quackery, and similar forms of swindling, and apparently generals, judges, and educated ladies require protection against the wily ones just as much as silly housemaids do.

### The Extirpation of Rabies.

Mr. Long was recently somewhat fiercely attacked in the House of Commons in regard to the muzzling order, but he held firmly to his position that the Government was bound to do its best to stamp out rabies. In dealing with the application of the muzzling order it was the policy of the Department to take the centre of the disease, as indicated by an outbreak of rabies, and draw around that centre a circle of 15 or 20 miles radius. This area was extended into other districts when it was possible to trace the course of a rabid dog into them, in doing which their inspectors had been very successful. By drawing a cordon round the area and imposing the muzzling order the district could be cleared of disease; but if sheep dogs were exempted the muzzling order would break down altogether, for every dog was a sheep dog if it was used at all, however occasionally, for driving sheep or cattle. The case of sporting dogs, however, was very different. They were a limited class, and were closely under control, and were watched and well cared for. In regard to the prospect of stamping out the disease he spoke very hopefully. In the quarter ending March 31st, 1896, there were 200 cases of rabies in the country; in the next quarter, 123; in the next, 67; in the next, 48; and in the quarter just ended the total number of actual cases reported had fallen to 6, in addition to which, however, there were 3 suspected cases. The policy adopted in dealing with rabies was one which was founded on a vast experience in the management of animal diseases, and he believed that, if they were able to carry it out, it would result in eradicating altogether this appalling disease. Mr. Vaughan Davies had a reasonable suggestion to make: "He believed that the only way to put down rabies and sheep-killing dogs was by making the owner answerable for everything the dog did." Good! But would not this be an even more serious task than the imposition of a muzzling order?

